# Improved bone bonding of hydroxyapatite spacers with a high porosity in a quantitative computed tomography‐image pixel analysis: A prospective 1‐year comparative study of the consecutive cohort undergoing double‐door cervical laminoplasty

**DOI:** 10.1002/jsp2.1080

**Published:** 2020-02-03

**Authors:** Yoshiki Takeoka, Takashi Yurube, Koichiro Maeno, Yutaro Kanda, Ryu Tsujimoto, Kunihiko Miyazaki, Yuji Kakiuchi, Shingo Miyazaki, Zhongying Zhang, Toru Takada, Kotaro Nishida, Minoru Doita, Ryosuke Kuroda, Kenichiro Kakutani

**Affiliations:** ^1^ Department of Orthopaedic Surgery Kobe University Graduate School of Medicine Kobe Japan; ^2^ Maeno Orthopaedic Clinic Himeji Japan; ^3^ Department of Orthopaedic Surgery Kobe Hokuto Hospital Kobe Japan; ^4^ Department of Orthopedic Surgery Graduate School of Medicine, University of the Ryukyus Okinawa Japan; ^5^ Department of Orthopaedic Surgery Iwate Medical University Graduate School of Medicine Iwate Japan

**Keywords:** bone bonding and absorption, cervical spine, computed tomography (CT), double‐door laminoplasty, hydroxyapatite spacer, porosity and pore size

## Abstract

Laminoplasty using hydroxyapatite (HA) spacers is widely performed in patients with cervical myelopathy. However, spacer dislocation is a critical complication caused by bone absorption and inadequate bone conductivity, and can result in dural damage and restenosis. We thus designed a prospective cohort study to clarify the feasibility of increased porosity HA spacers for double‐door laminoplasty by analyzing computed tomography (CT) images. Forty‐seven patients underwent cervical laminoplasty. Two different types of CERATITE HA spacer were used, either high porosity (50%) or low porosity (35%). These HA spacers were placed in an alternating manner into the laminae in each patient. In total, 85 high‐porosity (50%) HA spacers and 84 low‐porosity (35%) HA spacers were implanted. At postoperative 2 weeks, 3 months, 6 months, and 1 year, CT images were obtained. In both groups, the percentage of bone‐bonding boundary area of the HA spacer in contact with laminae and bone volume of the spinous process relative to the 2‐week value were calculated by a 3D and 2D CT‐image pixel analysis. The bone‐bonding ratio was significantly higher in high‐porosity (50%) than low‐porosity (35%) HA spacers at 3 months and thereafter (1 year, 69.3 ± 27.8% and 49.7 ± 32.9% respectively, *P* < .01). The bone volume in both groups significantly decreased with time (1 year, 73.2 ± 29.8% and 69.0 ± 30.4% respectively, *P* < .01), indicating bone absorption. This showed no significant difference between the HA spacers (*P* = .15) but was higher in high‐porosity (50%) than low‐porosity (35%) HA spacers throughout the study period. Meanwhile, spacer breakage was found in 4.7% of high‐porosity (50%) HA spacers and 1.2% of low‐porosity (35%) HA spacers (*P* = .37). In summary, high‐porosity (50%) HA spacers have the advantages of accelerated bone bonding and relatively decelerated bone absorption compared to low‐porosity (35%) HA spacers; however, possibly more frequent breakage of HA spacers with a high porosity (50%) requires careful, extended postoperative follow‐up.

## Graphical abstract

Computed tomography (CT)‐image pixel‐based bone‐bonding and bone‐volume analysis was performed in high‐porosity (50%) and low‐porosity (35%) hydroxyapatite (HA) spacers for cervical laminoplasty. A, Helical CT scanning was performed at postoperative 2 weeks, 3 months, 6 months, and 1 year. B, Three‐dimensional (3D) images were reconstructed from the Digital Imaging and Communications in Medicine (DICOM®) data. C, Based on these 3D images, HA spacers were extracted. D, The gap between the inserted HA spacer and split spinous processes was extracted to determine the spacer‐bound area to the bone. E, The boundary between the HA spacer and bone was further extracted as the two‐dimensional (2D) image, pixel in which was counted. Thereafter, the ratio of spacer‐bone contact area was calculated as the bone‐bonding ratio to assess fusion between the spacer and spinous processes. F, The bone volume of the dorsal parts in the split spinous processes in contact with the spacer was measured to assess the progression of time‐dependent bone absorption around HA.

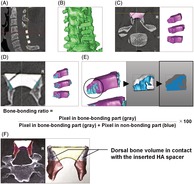

## 1 INTRODUCTION

Cervical myelopathy occurs for spondylosis, ossification of posterior longitudinal ligament (OPLL), spinal trauma, and other diseases. Patients have a variety of symptoms, such as spastic gait, hand clumsiness, muscular atrophy, sensory disturbance, and sphincter disturbance. Cervical laminoplasty is a standard surgical procedure and widely performed in patients with cervical myelopathy. At present, the methods of cervical laminoplasty are divided into two types by the site of osteotomy: double‐door type^1^ and open‐door type.^2^ Double‐door laminoplasty was first described in 1982,^1^ and can be recommended today owing to the lower risk of postoperative C5 palsy compared to open‐door laminoplasty.^3^ While autologous bone^4^ and artificial materials^5^ have been used to maintain the enlarged spinal canal, the use of hydroxyapatite (HA) spacers has become popular because of the benefits of no donor site pain, shorter operative time, and decreased blood loss.^6^ However, several complications have been reported including the dislocation of the HA spacer into the canal resulting in dural damage and restenosis.^7^, ^8^, ^9^ Bone absorption around HA leads to shortening of the split spinous processes and dislocation of the spacers, which is possibly caused by inadequate bone conductivity.^6^ The bone bonding and osteoconductive capability of the material, as well as the contact between the spacer and spinous processes, are essential to maintain the enlarged spinal canal and reduce the complications. Although bone bonding between the HA spacer and spinous processes have been examined with computed tomography (CT),^10^, ^11^, ^12^, ^13^ it is difficult to evaluate bony fusion in objective, quantitative manners. In addition, only a few studies have been conducted prospectively.^13^

The porosity of HA is related to its osteoconductive capability and mechanical strength, as HA with an increased porosity has a high osteoconductive capability^14^ but also a relatively high risk of breakage.^15^ However, no prospective studies have compared bone bonding and absorption in HA spacers with different types of porosity for cervical laminoplasty. The aim of the current study was to clarify the utility of increased porosity HA spacers for double‐door laminoplasty by analyzing CT images.

## 2 MATERIALS AND METHODS

### 2.1 Study design

Prospective 1‐year follow‐up study of the consecutive cohort who underwent cervical laminoplasty for the comparison of HA spacers with two different types of porosity. Level of evidence: Level 2.

### 2.2 Ethics statement

All human experiments were performed under the approval and guidance of the Institutional Review Board at Kobe University Graduate School of Medicine (No. 829). Written informed consent was obtained from each patient in accordance with the principles of the Declaration of Helsinki and the laws and regulations of Japan.

### 2.3 Patients

From January 2005 to August 2008, 47 consecutive patients who underwent double‐door laminoplasty for cervical myelopathy at the authors' institution were enrolled in this study, and prospectively followed up for at least 1 year after surgery. The subjects included 29 men and 18 women, and the mean age was 65.2 ± 9.3 [40–84] years. The preoperative diagnoses were 38 cases of spondylosis, 7 cases of OPLL, and 2 cases of disc herniation. Patients with a requirement of instrumentation and fusion, for example, rheumatoid arthritis, were excluded. Patients with malignancy and those with diabetes mellitus of a glycated hemoglobin (HbA1c) level of 8.0% or more were also excluded. In 169 laminae of these 47 patients, CT images were obtained at postoperative 2 weeks, 3 months, 6 months, and 1 year. All patients completed a minimum 1‐year clinical and radiological follow‐up (follow‐up rate: 100.0%). The mean follow‐up period was 380 ± 29.8 days.

### 2.4 HA spacers

**Figure 1:**
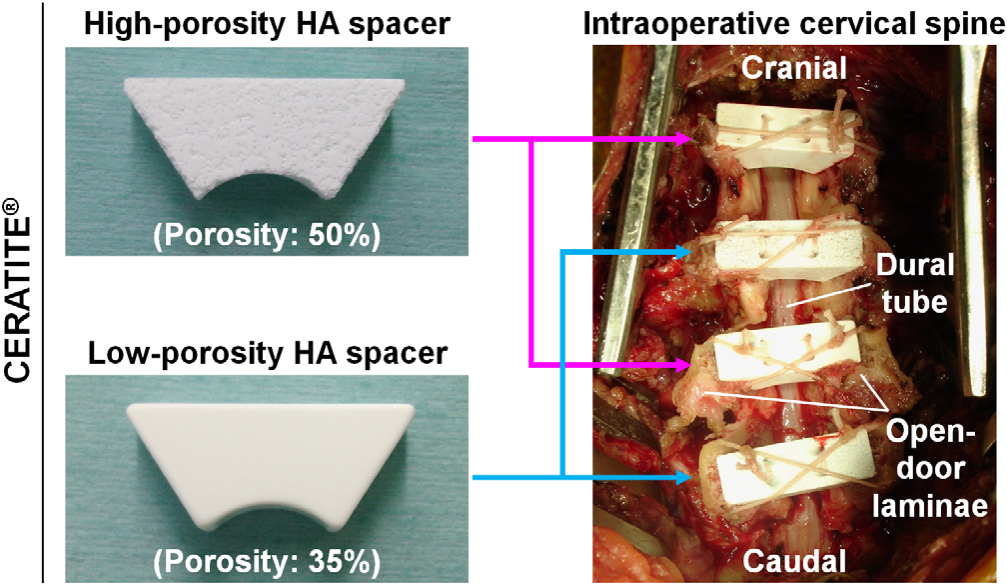
High‐porosity (50%) and low‐porosity (35%) hydroxyapatite (HA) spacers for cervical laminoplasty. Two different types of HA spacers, CERATITE (NGK Spark Plug Co., Ltd., Aichi, Japan), were used in this study, consisting of 70% hydroxyapatite and 30% β‐tricalcium phosphate with a large pore and high porosity (50%) and with a small pore and low porosity (35%). During laminoplasty in the cervical spine, spinous processes were vertically split using a threaded‐wire saw, and these HA spacers were placed between the split spinous processes. High‐porosity (50%) and low‐porosity (35%) HA spacers were placed in an alternating manner. In total, 85 high‐porosity (50%) HA spacers and 84 low‐porosity (35%) HA spacers were implanted in 47 patients with cervical spondylotic myelopathy. Nonabsorbable sutures were used to anchor the spacers to the split spinous processes

Two different types of clinically available HA spacers, CERATITE (NGK Spark Plug Co., Ltd., Aichi, Japan), were used in this study, that consisted of 70% HA and 30% β‐tricalcium phosphate: a large‐pore, high‐porosity HA spacer (porosity, 50%; pore diameter, 170 μm; bending strength, 60 kgf/cm^2^; compressive strength, 100 kgf/cm^2^; interpore path diameter, 60 μm; firing temperature, 1300°C) and a low‐porosity HA spacer (porosity, 35%; pore diameter, 5 μm; bending strength, 400 kgf/cm^2^; compressive strength, 1600 kgf/cm^2^; interpore path diameter, 0.5 μm; firing temperature, 1100°C) (Figure 1). Technically, polymer beads were mixed in the high‐porosity (50%) HA, which develop large pores upon burned.

### 2.5 Surgery

Under general anesthesia in the prone position, spinous processes were vertically split using a threaded‐wire saw^16^ at C2–C6, C3–C6, C3–C7, or C4–C7, depending on the patients' neurological and radiological findings, and HA spacers were placed between the split spinous processes (Figure 1). The spacer was not applied for the level with foraminotomy whose lamina had been removed. From January 2005 to November 2006, high‐porosity (50%) HA spacers were placed in the odd laminae (C3, C5, and C7) and low‐porosity (35%) HA spacers in the even laminae (C2, C4, and C6) (87 laminae in 24 cases). From December 2006 to August 2008, high‐porosity (50%) HA spacers were placed in the even laminae, and low‐porosity (35%) HA spacers were placed in the odd laminae (82 laminae in 23 cases). In total, 85 high‐porosity (50%) HA spacers and 84 low‐porosity (35%) HA spacers were implanted. The size of HA spacers was determined based on the intraoperative adaptability between the split spinous processes. Nonabsorbable sutures were used to anchor the spacers to the split spinous processes.

### 2.6 Clinical evaluation

For clinical evaluation for cervical myelopathy, the scoring system developed by the Japanese Orthopaedic Association (JOA) (total 17 points, 1994 revised edition)^17^ was applied and measured before and 1 year after surgery, and functional improvements in the JOA score were expressed as the recovery rate: (postoperative score − preoperative score)/(17 − preoperative score) × 100.^18^

### 2.7 CT‐image pixel analysis

**Figure 2:**
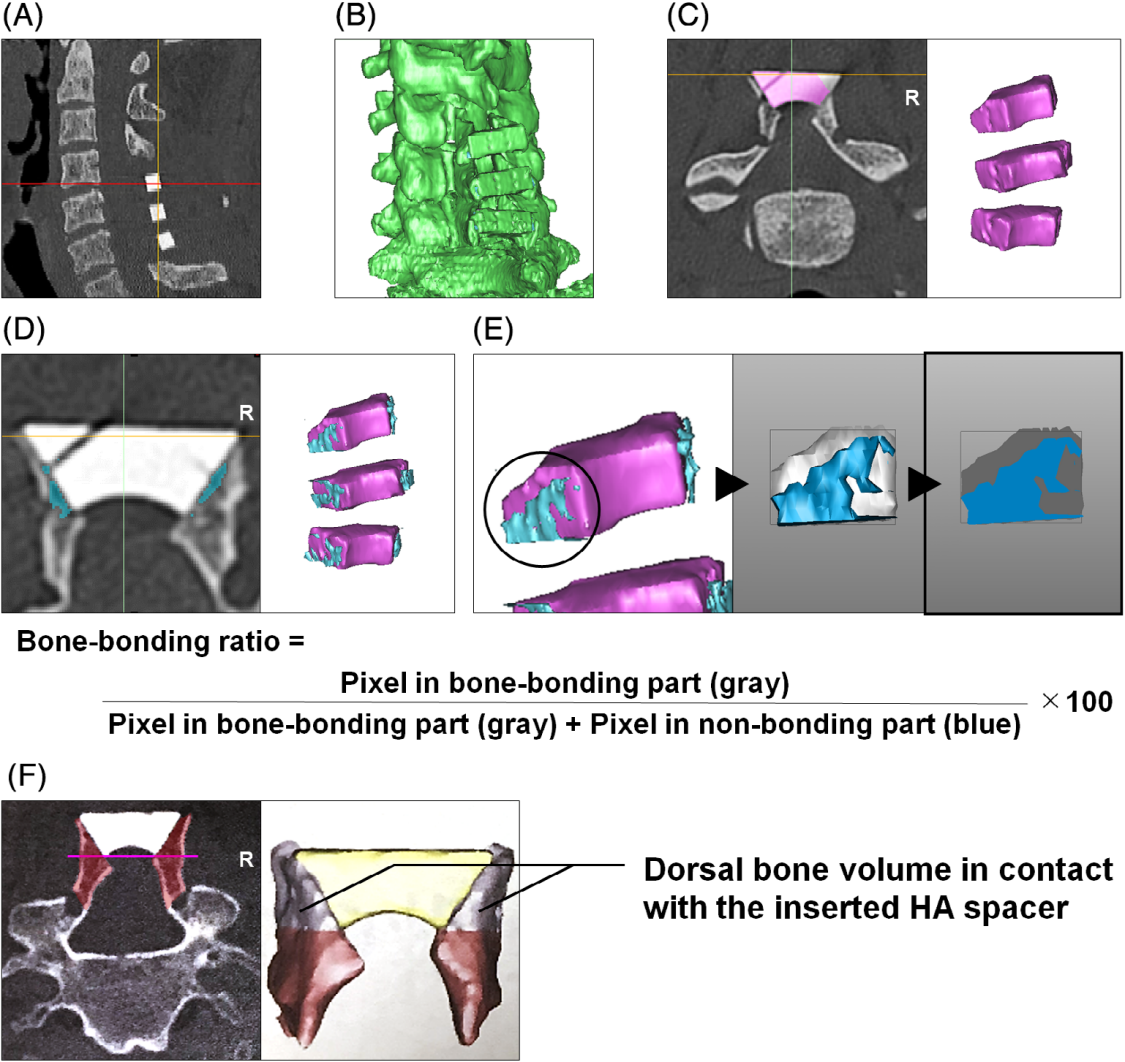
Computed tomography (CT)‐image pixel‐based bone‐bonding and bone‐volume analysis of high‐porosity (50%) and low‐porosity (35%) hydroxyapatite (HA) spacers for cervical laminoplasty. A, Helical CT scanning was performed at postoperative 2 weeks, 3 months, 6 months, and 1 year. B, Three‐dimensional (3D) images were reconstructed from the Digital Imaging and Communications in Medicine (DICOM®) data. C, Based on these 3D images, HA spacers were extracted. D, The gap between the inserted HA spacer and split spinous processes was extracted to determine the spacer‐bound area to the bone. E, The boundary between the HA spacer and bone was further extracted as the two‐dimensional (2D) image, pixel in which was counted. Thereafter, the ratio of spacer‐bone contact area was calculated as the bone‐bonding ratio to assess fusion between the spacer and spinous processes. F, The bone volume of the dorsal parts in the split spinous processes in contact with the spacer was measured to assess the progression of time‐dependent bone absorption around HA

Helical CT scans were performed (Figure 2A), and the Digital Imaging and Communications in Medicine (DICOM®) data were reconstructed into three‐dimensional (3D) images with Materialise Mimics (Materialise Naamloze Vennootschap, Leuven, Belgium) (Figure 2B). Based on these 3D images, HA spacers were extracted with the threshold of CT number at 700 Hounsfield unit (HU) based on a higher density and CT number of CERATITE® than of human bone (Figure 2C). Then, the gap between each spacer and the split spinous processes was extracted to determine the area where the spacer was not bound to the bone. The threshold of CT number for the bone was set at 226 HU, in which the area with the CT number of less than 226 HU was regarded as a gap (Figure 2D). Then, the boundary between the spacer and bone was extracted as the two‐dimensional (2D) image, and the 2D pixel in each area was counted using Geomagic Studio (Geomagic, Inc., Morrisville, North Carolina). Thereafter, the ratio of spacer‐bone contact area was calculated using the following formula: bone‐bonding ratio = 100 × pixel number in bone‐bonding part/(pixel number in bone‐bonding part + pixel number in nonbonding part). The gap disappearance between the HA spacer and split spinous processes was automatically detected, which was recognized as a condition of completed bone bonding^13^, ^19^ (Figure 2E). Furthermore, to investigate time‐dependent bone absorption of the laminae, the bone volume of the dorsal part in the split spinous processes in contact with the HA spacer, without the inclusion of newly synthesized bone at the gutter of laminae, was measured at each time point using Materialise Mimics (Figure 2F). The bone volume at each time point was expressed as a percentage relative to the value of high‐porosity (50%) HA spacers at postoperative 2 weeks. In addition, the presence of broken HA spacers was counted at each time point. In this study, three independent researchers evaluated the bone‐bonding ratio at each follow‐up period to calculate the inter‐observer reliability.

### 2.8 Statistical analysis

Data were expressed as the mean ± SD. The paired *t* test was used to compare preoperative and postoperative JOA scores for cervical myelopathy. Two‐way repeated measures ANOVA with the Scheffe post hoc test was used to assess effects of *treatment (spacer type)* and *time (postoperative follow‐up period)* in bone bonding and absorption. The Fisher exact probability test was also applied to assess the rate of HA spacer breakage. In addition, intra‐class correlation coefficient was calculated to determine the inter‐observer reliability for the bone‐bonding ratio. The *P*‐values <.05 were regarded as statistically significant using IBM SPSS Statistics 23.0 (IBM, Armonk, New York).

## 3 RESULTS

### 3.1 Successful surgical treatment with an acceptable postoperative neurological recovery

All patients received double‐door laminoplasty surgery and postoperative minimum 1‐year follow‐up without serious adverse events. The mean JOA score significantly improved from 10.5 at preoperation to 13.9 at postoperative 1 year (*P* < .01). The mean recovery rate was 52.3%. No postoperative infection was found, including redness around the scar, abnormal blood test results, and high fever.

### 3.2 Accelerated bone bonding of high‐porosity (50%) HA spacers than of low‐porosity (35%) HA spacers for cervical laminoplasty

**Figure 3:**
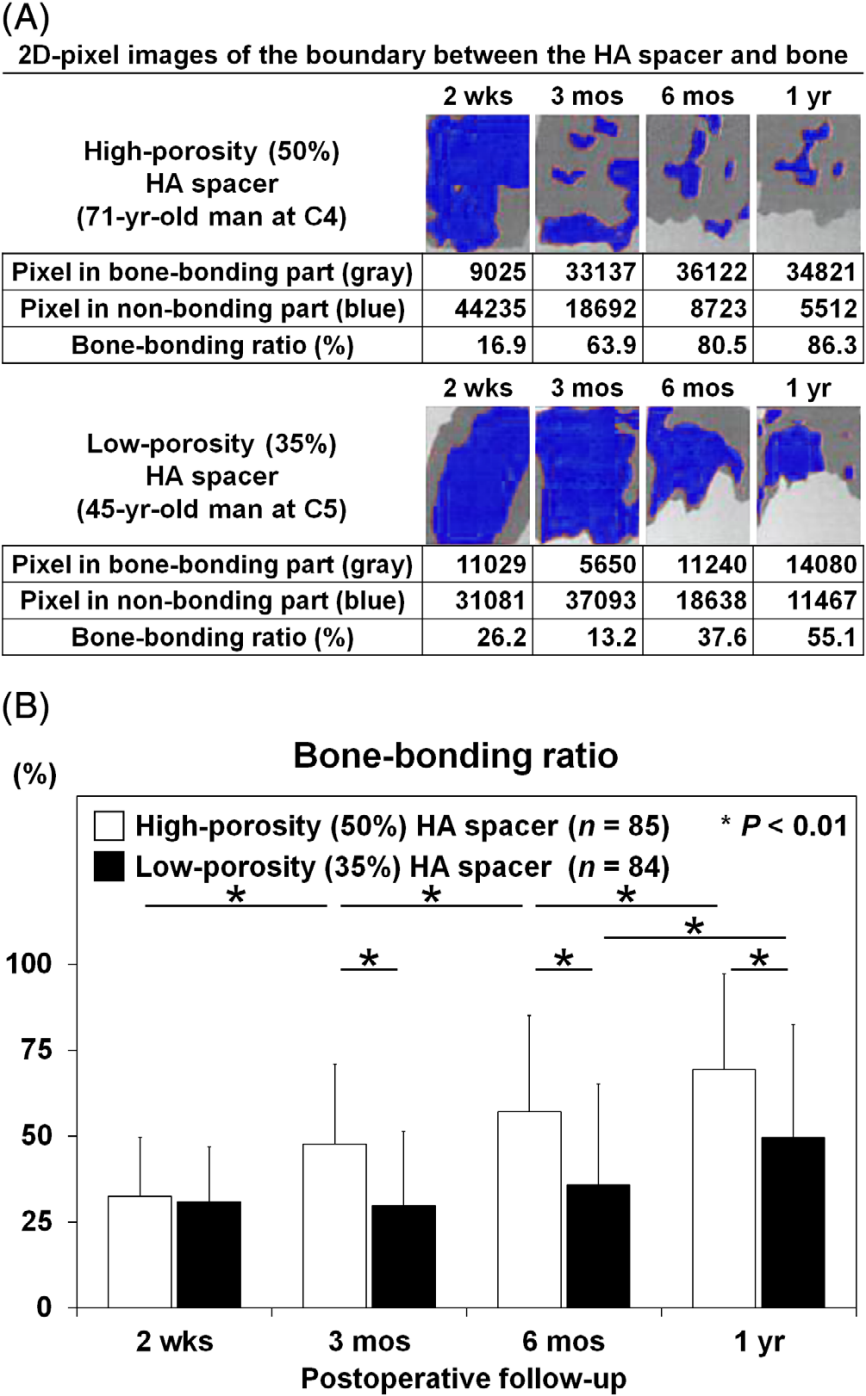
Accelerated bone bonding of high‐porosity (50%) hydroxyapatite (HA) spacers than of low‐porosity (35%) HA spacers for cervical laminoplasty. A, Representative two‐dimensional (2D)‐pixel images of the boundary between the HA spacer and bone in a 71‐year‐old male patient with a high‐porosity (50%) HA spacer at C4 and in a 45‐year‐old male patient with a low‐porosity (35%) HA spacer at C5. Pixel numbers in bone‐bonding and nonbonding parts were measured, and the bone‐bonding ratio was calculated. B, The bone‐bonding ratio of high‐porosity (50%) and low‐porosity (35%) HA spacers at postoperative 2 weeks (2 wks), 3 months (3 mos), 6 months (6 mos), and 1 year (1 yr). The bone‐bonding ratio was significantly higher in high‐porosity (50%) HA spacers at postoperative 3 months, 6 months, and 1 year compared to in low‐porosity (35%) HA spacers (all *P* < .01). Furthermore, the bone‐bonding ratio of high‐porosity (50%) HA spacers significantly increased at postoperative 3 months and thereafter (all *P* < .01), while that of low‐porosity (35%) HA spacers showed a significant increase only between postoperative 6 months and 1 year (*P* < .01). Data are the mean ± SD. Two‐way repeated measures ANOVA with the Scheffe post hoc test was used

Representative 2D images and calculated data of the boundary between the inserted HA spacer and split spinous processes in both study groups are shown in Figure 3A. In our CT‐image pixel analysis, the bone‐bonding ratios of high‐porosity (50%) HA spacers and low‐porosity (35%) HA spacers were 32.5 ± 17.1% and 30.9 ± 16.1% at postoperative 2 weeks, 47.8 ± 23.2% and 29.7 ± 21.7% at postoperative 3 months, 57.2 ± 27.9% and 35.9 ± 29.3% at postoperative 6 months, and 69.3 ± 27.8% and 49.7 ± 32.9% at postoperative 1 year, respectively. The ratio was significantly higher in high‐porosity (50%) HA spacers at postoperative 3 months and thereafter compared to in low‐porosity (35%) HA spacers (all *P* < .01). Furthermore, the ratio of high‐porosity (50%) HA spacers showed a time‐dependent increase with statistical significance at all time points (all *P* < .01), while that of low‐porosity (35%) HA spacers significantly increased only between postoperative 6 months and 1 year (*P* < .01) (Figure 3B). For CT‐based bone‐bonding analysis, the inter‐observer reliability was 0.924 at postoperative 2 weeks, 0.942 at postoperative 3 months, 0.929 at postoperative 6 months, and 0.872 at postoperative 1 year by intraclass correlation coefficient, all values of which indicated an acceptable reproducibility by three examiners.

### 3.3 Relatively decelerated bone absorption of high‐porosity (50%) HA spacers than of low‐porosity (35%) HA spacers for cervical laminoplasty

**Figure 4:**
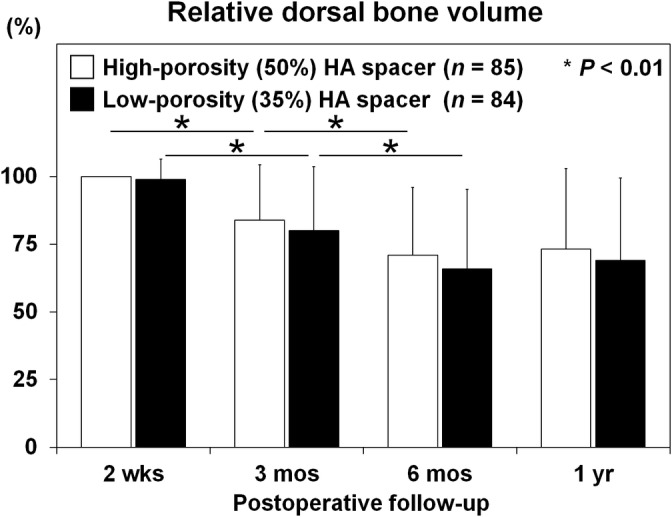
Relatively decelerated bone volume of high‐porosity (50%) hydroxyapatite (HA) spacers than of low‐porosity (35%) HA spacers for cervical laminoplasty. The dorsal bone volume in contact with high‐porosity (50%) and low‐porosity (35%) HA spacers at postoperative 2 weeks (2 wks), 3 months (3mos), 6 months (6 mos), and 1 year (1 yr). The dorsal bone volume in contact with high‐porosity (50%) and low‐porosity (35%) HA spacers both significantly decreased at postoperative 3 and 6 months (all *P* < .01). The bone volume did not show any significant difference between the two types of HA spacers at postoperative 3 months (*P* = .12), 6 months (*P* = .20), and 1 year (*P* = .29); however, higher percentages in the bone volume of high‐porosity (50%) HA spacers than of low‐porosity (35%) HA spacers were observed throughout the study period. The volume at each time point was expressed as the relative value of high‐porosity (50%) HA spacers at postoperative 2 weeks (100.0%). Data are the mean ± SD. Two‐way repeated measures ANOVA with the Scheffe post hoc test was used

The dorsal bone volumes in contact with high‐porosity (50%) and low‐porosity (35%) HA spacers were 83.8 ± 20.5% and 80.1 ± 23.6% at postoperative 3 months, 70.9 ± 25.1% and 66.0 ± 29.8% at postoperative 6 months, and 73.2 ± 29.8% and 69.0 ± 30.4% at postoperative 1 year, respectively. As expected, time‐dependent decreases in the dorsal bone volume were observed commonly between the two types of the HA spacer (both overall *P* < .01: both *P* < .01 at postoperative 3 months and both *P* < .01 at postoperative 6 months). Although the bone volume did not show any significant difference in both experimental groups at all time points (overall *P* = .15: *P* = .12 at postoperative 3 months, *P* = .20 at postoperative 6 months, and *P* = .29 at postoperative 1 year), higher percentages in the bone volume of high‐porosity (50%) HA spacers than of low‐porosity (35%) HA spacers were observed throughout the study period (Figure 4).

### 3.4 Possibly more frequent breakage of high‐porosity (50%) HA spacers than of low‐porosity (35%) HA spacers for cervical laminoplasty

**Figure 5:**
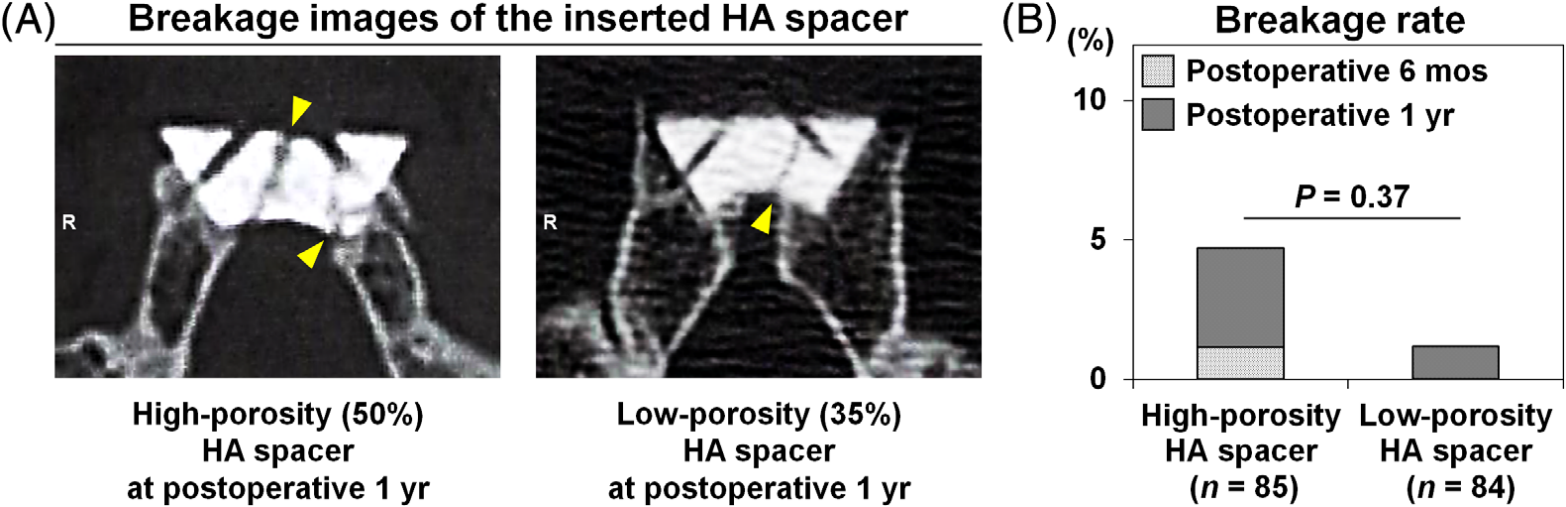
Possibly more frequent breakage of high‐porosity (50%) hydroxyapatite (HA) spacers than of low‐porosity (35%) HA spacers for cervical laminoplasty. A, Representative computed tomography images of the breakage of the inserted high‐porosity (50%) and low‐porosity (35%) HA spacers are shown. Yellow arrows indicate broken cracks. B, In total, 5 damaged HA spacers were observed in 47 patients at postoperative 1 year, including 4 of 85 high‐porosity (50%) HA spacers (4.7%) and 1 of 84 low‐porosity (35%) HA spacers (1.2%). Of the 4 damaged high‐porosity (50%) HA spacers, 1 was recognized at postoperative 6 months whereas 3 were at postoperative 1 year after surgery. Meanwhile, 1 of low‐porosity (35%) HA spacers was damaged at postoperative 1 year after surgery (6 months, *P* > .99; 1 year, *P* = .37). No cases with the dislocation of HA spacers were found in both experimental groups. The Fisher exact probability test was used

Damage in 5 HA spacers was observed during the 1‐year follow‐up period, including 4 of 85 (4.7%) high‐porosity (50%) HA spacers and 1 of 84 (1.2%) low‐porosity (35%) HA spacers (Figure 5). Of the 4 damaged high‐porosity (50%) HA spacers, 1 and 3 were recognized at postoperative 6 months and 1 year, respectively. Meanwhile, only a breakage of low‐porosity (35%) HA spacers was found at 1 year after surgery (6 months, *P* > .99; 1 year, *P* = .37). However, no dislocations of the damaged spacers were detected. No postoperative neurological deficits were observed. Consequently, no additional surgery was required.

## 4 DISCUSSION

This is the first prospective study to demonstrate the feasibility of HA spacers with a high porosity (50%) compared to with a low porosity (35%) for double‐door laminoplasty in patients with cervical myelopathy. Although prior studies have analyzed bone bonding and/or fusion between the HA spacer and laminae subjectively based on the gap disappearance^13^, ^19^ or the Ichikawa bone‐bonding classification,^7^, ^10^, ^11^, ^20^ we used a more objective, quantitative CT‐based method to assess bone bonding and absorption. In addition, this study's prospective design is advantageous to reduce bias compared to other retrospective studies,^10^, ^11^, ^12^ as published prospective studies have still been limited.^13^ Additional efforts to shrink the deviation of spacer‐implanted segments as well as the individual variability are also noteworthy in this study. This study's comparison between the two alternating HA spacers in each patient is useful to minimize the association of metabolic disorders, for example, diabetes and osteoporosis, in elderly patients and also the difference among baseline pathologies for cervical myelopathy. In literature, higher‐porosity HA spacers have a trend of higher bone bonding: 27.5%–83.9% in spacers with a porosity of 40%,^19^, ^20^, ^21^ 8.7%–60.5% with a porosity of 60%,^7^, ^10^ and 67.0%–90% with a porosity of 75%.^11^, ^13^ In the current study, high‐porosity (50%) HA spacers showed an accelerated bone bonding compared to low‐porosity (35%) HA spacers, which is in agreement with reported evidence. Then, although the bone volume of laminae in contact with the inserted HA spacer showed a trend of time‐dependent absorption both in high‐porosity (50%) and low‐porosity (35%) HA spacers, a relatively reduced severity of bone absorption was found in high‐porosity (50%) HA spacers compared to in low‐porosity (35%) HA spacers. Bone absorption is much more commonly observed in laminoplasty with HA spacers than with autologous bone grafts, possibly because of inadequate contact and fixation between the spacer and split spinous processes.^6^ Therefore, the use of high‐porosity (50%) HA spacers has the advantages of accelerated bone bonding and relatively decelerated bone absorption in cervical laminoplasty.

Higher‐porosity HA spacers have been reported to have a high breakage rate, ranging from 2.9% to 21.3%.^8^, ^10^, ^11^, ^13^ In this study, the breakage rate was 4.7% in high‐porosity (50%) HA spacers and 1.2% in low‐porosity (35%) HA spacers. No dislocations or neurological complications were observed. However, sagittal displacement of HA spacers has been reported to occur in 41.7%,^7^ while additional surgeries were required in 3.1% due to dural laceration and neurological deterioration.^8^ Therefore, careful attention and extended follow‐up are required.

This study has several limitations. First, the follow‐up period was only 1 year, which is potentially too short to assess bone bonding and absorption because bony fusion may not complete within 1 year. Accelerated bone bonding within early postoperative periods is our primary interest; however, bone bonding and absorption can change more than 1 year after surgery. In this study, CT‐based follow‐up at postoperative 2 years was not allowed because of the study budget constraints and also patient radiation exposure issues. Second, the bone‐bonding ratio does not directly indicate mechanical strength. Our measurement to assess the contact between the HA spacer and spinous process might not be equivalent to bony fusion as fusion should not be recognized as early as at postoperative 2 weeks. Third, the inclusion of C2 and C7 is controversial based on the different biomechanics and possibly altered fusion rate. Finally, our data are slightly dated, as it has taken time to analyze and comprise the results. However, CERATITE is widely used even today in Japan; therefore, this study should provide useful information for the selection of porosity in HA spacers and also for the development of new bioceramic materials.

In conclusion, for the treatment of cervical myelopathy by double‐door laminoplasty, high‐porosity (50%) HA spacer is feasible and offers the advantages of accelerated bone bonding and relatively decelerated bone absorption compared to low‐porosity (35%) HA spacers. However, spacer breakage could be a possible issue in laminoplasty using high‐porosity (50%) HA spacers, which requires careful, extended postoperative follow‐up.

## CONFLICT OF INTEREST

The authors have no conflicts of interest to declare.

## AUTHOR CONTRIBUTIONS

Y.T., T.Y., K.M., K.N., M.D., and K.K. designed the study; Y.T., T.Y., and K.M. performed the data collection and analysis; Y.T., T.Y., K.M., Y.Kanda, R.T., K.M., Y.Kakiuchi, S.M., Z.Z., T.T., K.N., M.D., R.K., and K.K. contributed to the data analysis and interpretation; Y.T., T.Y., and K.M. wrote the manuscript; and Y.Kanda, R.T., K.M., Y.Kakiuchi, S.M., Z.Z., T.T., K.N., M.D., R.K., and K.K. revised it critically for important intellectual content. All authors approved the final version to be published.
